# A mobile app (IDoThis) for multiple sclerosis self-management: development and initial evaluation

**DOI:** 10.1186/s12911-022-02078-z

**Published:** 2022-12-13

**Authors:** Zeinab Salimzadeh, Shahla Damanabi, Reza Ferdousi, Sheida Shaafi, Leila R. Kalankesh

**Affiliations:** 1 Department of Health Information Technology, School of Management and Medical Informatics, Tabriz University of Medical Sciences, Daneshgah Ave, Tabriz, Iran; 2 Health Care Services Management Research Center, Tabriz University of Medical Sciences, Tabriz, Iran; 3 Research Center of Psychiatry and Behavioural Sciences, Tabriz University of Medical Sciences, Tabriz, Iran; 4 Department of Neurology, Tabriz University of Medical Sciences, Tabriz, Iran

**Keywords:** Multiple sclerosis, Mobile applications, Self-management, Mobile health, M-Health

## Abstract

**Background:**

Multiple sclerosis (MS) is one of the most common neurological disorders worldwide, and self-management is considered an essential dimension in its control. This study aimed to develop an evidence-based mobile application for MS self-management and evaluate it.

**Methods:**

This study was undertaken in three phases: content preparation, design, and evaluation. In the content preparation phase, the researchers extracted MS self-management needs based on related guidelines and guides, existing apps on the self-management of MS, and the field experts' views and confirmation. The design phase was conducted in five steps: defining app functionalities, depicting the wireframe, preparing the media, coding the app, and testing the app’s performance. The app was developed using the Android Studio environment and Java programming language for the Android operating system. The performance of the developed app was tested separately in several turns, and existing defects were corrected in each turn. Finally, after using the app for three weeks, the app was evaluated for its short-term impact on MS management and user-friendliness using a researcher-constructed questionnaire from participants’ (N = 20) perspectives.

**Results:**

The IDoThis app is an offline app for people with MS that includes five main modules: three modules for training or informing users about different aspects of MS, one module for monitoring the user's MS condition, and a reporting module. In the initial evaluation of the app, 75% (n = 15) of participants mentioned that using this app improved MS self-management status at intermediate and higher levels, but 25% (n = 5) of the participants mentioned that the effect of using the app on the self-management tasks was low or was very low. The majority of users rated the user-friendliness of the app as high. The users found the sections “exercises in MS” and “monitoring of MS status” beneficial to their self-management. Still, the fatigue and sleep management sections are needed to meet users' expectations.

**Conclusion:**

Using IDoThis app as a self-management tool for individuals with MS appears feasible, that can meet the need for a free and accessible self-management tool for individuals with MS. Future directions should consider the users’ fatigue and sleep management expectations.

**Supplementary Information:**

The online version contains supplementary material available at 10.1186/s12911-022-02078-z.

## Background

Self-management originates from the idea that individuals with a chronic condition should take an active, central role in managing their diseases, secondary problems, health care, and the day-to-day monitoring of chronic conditions [1]. MS is one of the chronic diseases of the central nervous system and one of the most common causes of disability in young people. Self-management is considered a principal task in its management and control. MS is associated with a wide range of symptoms. The presence and complexity of MS symptoms complicate its management, which can become an exhausting task for individuals with MS [2–8].

Due to the nature of MS, its self-management is critical, multifaceted, and individual. MS self-management includes elements of the general self-management framework with specific attention to MS in each person, obtained from the unique experience of living with MS in each person [2–8]. Successful self-management of MS disease requires multiple tasks as follows:

1. learning about the disease and its symptoms and treatments
2. monitoring health status
3. choosing a healthy lifestyle
4. creating short-term and long-term self-management goals
5. managing mental status
6. developing support networks access to health care providers supporting self-management
7. managing fatigue through planning and prioritizing activities
8. reconstructing cognition to deal with anger and frustrations of living with MS [8, 9].

There are guidelines and guides for the management of MS developed by reputable MS-related organizations and institutions such as National Multiple Sclerosis Society, MS Trust, Multiple Sclerosis International Federation, Multiple Sclerosis Association of America, Multiple Sclerosis Society of Canada, Multiple Sclerosis Western Australia, European MS Platform, Multiple Sclerosis Foundation, and the Consortium of Multiple Sclerosis [10–18]. However, using these guidelines in everyday life can be difficult for people with MS. Development of self-management mobile apps based on such guidelines can be an effective solution to encourage, help and adhere to self-management [19–22].

In recent years, mobile apps with different goals have been developed for MS, and several studies have reviewed the existing apps. These studies have highlighted limitations of the apps in terms of their functionality, their supply and demand mismatch, their failure to meet some of the self-management needs, the gap in the collaboration of patients and health care providers in apps development, and the current gap between MS self-management needs and existing apps [19, 20, 23–26]. In our previous study, the self-management needs of people with MS were elicited from a series of semi-structured interviews, which revealed the themes ranging from information needs of people with MS (awareness of MS from reliable sources, solutions for adaptation to MS, awareness of valuable exercises and beneficial foods in MS), aspects that should be monitored for MS management (MS symptoms, rest status, and stress status), and communications needs of people with MS (communication with specialists and optimistic people with MS) [27]. Also, results from our other study characterized several apps developed for MS (n = 104) with a variety of purposes ranging from diagnosis to treatment, tests, self-management, communication for MS patients, awareness-raising, access to journals and news, conferences & meetings, and supporting & donating to the MS community [28]. Despite the considerable number of apps (n = 27) for MS self-management, they needed to be more comprehensive in fully meeting the needs of those with MS due to the wide range of MS symptoms. Given the importance of self-management in MS and despite the positive evaluation of some existing MS apps in the studies [29–36], there is a need to develop apps to cover the essential aspects of MS self-management. Therefore, this study was conducted to design a mobile application in Persian language for MS self-management and evaluate it primarily.

## Methods

**Fig. 1:**
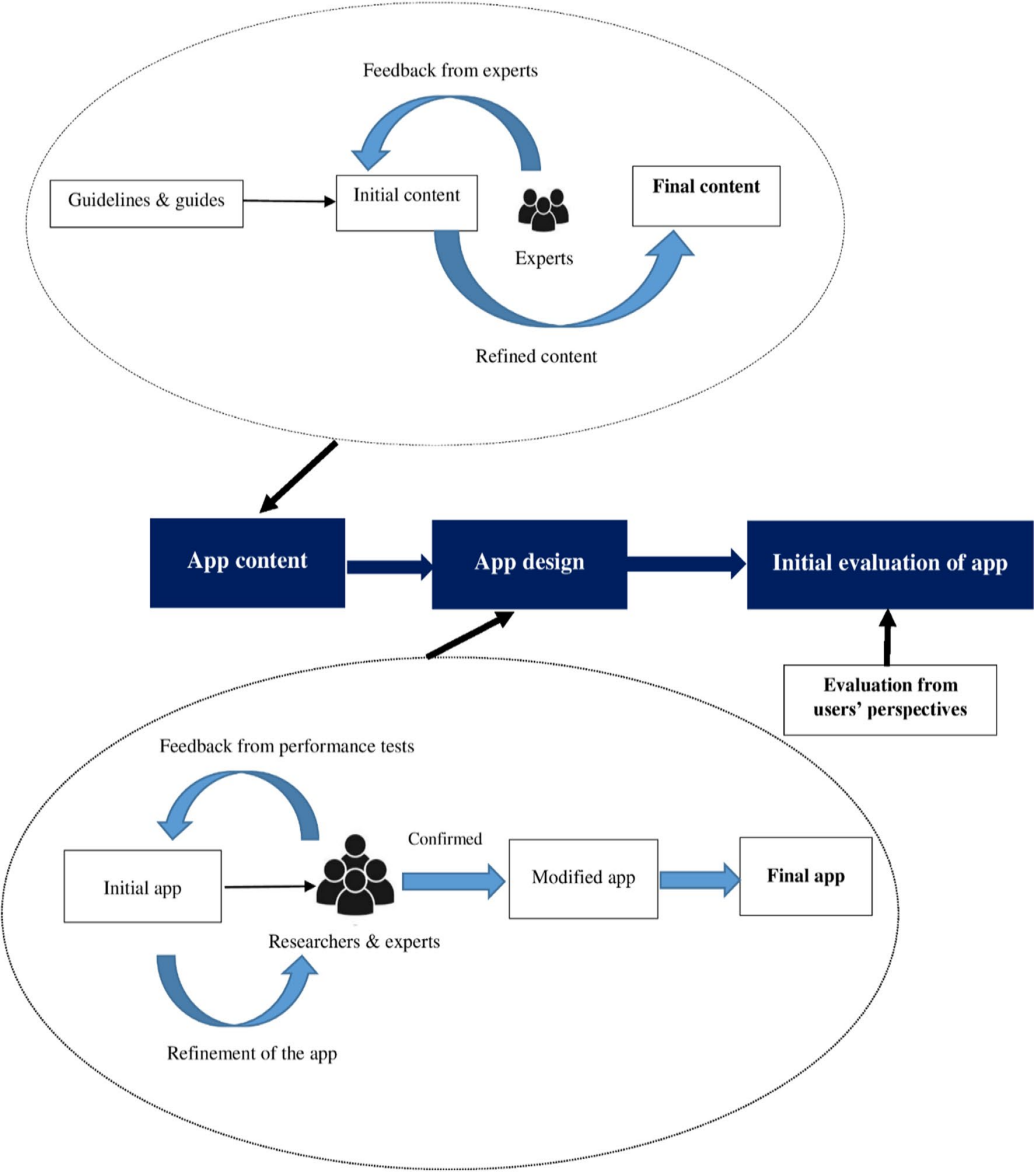
A schematic view of three phases for the development of MS self-management (IDoThis) app

The present study aims to develop and evaluate a self-management mobile application for individuals with MS. This study was conducted in three phases:

1. Determining the MS self-management needs (content preparation phase)
2. Designing a self-management mobile application (app design phase)
3. Evaluating the app from users’ perspectives (app evaluation phase) (Fig. 1).

### Phase 1: Content preparation

Mobile content is defined as any form of media (pictures, music, text, videos) contained in mobile apps for the mobile device (cell phone or tablet) [37]. The initial content was prepared after extracting MS self-management needs based on the information available in guidelines and guides, paying attention to the results of our two previous studies in the field of existing apps for MS self-management and extracting needs by interviewing MS patients [27, 28].

First, the researchers searched websites of MS international organizations and accredited associations to retrieve MS self-management authoritative guides and guidelines. Then they extracted available resources [10–18]. The resources were reviewed to achieve the MS self-management framework.

Considering various dimensions of MS self-management (having awareness about MS, monitoring MS status, having a healthy lifestyle, managing fatigue, managing mental status, setting short-term and long-term self-management goals, developing support networks, and restructuring cognition) and the impossibility of focusing on all dimensions in one app, the researchers decided to design application concentrating on four dimensions of MS self-management, ranging from awareness of MS to healthy lifestyle in MS (nutrition and exercise in MS), MS monitoring, and fatigue management. First, the researchers (ZS and SS) reviewed the existing guidelines and guides in the four dimensions. The preliminary content was extracted after a comparative study of the different guidelines and guides. In disagreements between various sources, the guidelines were given priority. All the selected guidelines and guides are listed in Additional file 1. Finally, the initial content was given to three specialists (one neurologist, one nutritionist, and one physiotherapist) to get expert opinions. The initial content was finalized after applying experts' opinions and getting their' confirmation.

### Phase 2: The mobile application design

The mobile app design phase was conducted in five steps:

- defining app functionalities
- depicting the app wireframe
- preparing the media
- coding the app
- testing the app’s performance.

### Defining functionalities of the mobile app

The expected and necessary functionalities of the app were determined and finalized according to the results of our previous studies [27, 28] and the final content of the app was defined. The use-case diagram was deployed to model, present, and analyze the application's essential functionalities using Rational Rose software version 8 and UML language.

### Depicting the wireframe of the mobile app

The researchers started the app design by depicting the app wireframe. The wireframe is a basic design or visual representation of a mobile application, website, system, or application before implementation. In this technique, the frame of the desired product is considered, and then with smooth lines and simple geometric shapes, the way of placing the components in it is determined. It is possible to produce wireframes in both paper and digital methods. Drawing a wireframe saves time, sets system priorities, improves content display, facilitates needed changes, and helps achieve better results [38]. The app's wireframe was sketched manually to show the app's different sections. Then the structure of the content in other parts of the application was also defined. Based on the drawn wireframe, five main sections were considered for the app and it was decided to use text, audio, and video formats in different parts of the app.

### Preparing the media

After finalizing the structure and key aspects of the content, the researchers prepared different elements and media for the app. These parts included: images, logo, texts, video and audio files. All images used for the app's main parts were manually drawn, painted, and digitized.

To make the app logo, we used the orange ribbon (as the awareness symbol of MS), a symbol of man and nerve, as the main components of the logo. Then, based on an initial idea, the initial design of the logo was drawn manually and was implemented using Adobe Photoshop cc 2017 software. In the next step, the opinions of a graphic expert were obtained about the initial design, and the required changes were applied.

iSpring suite 8 was used to prepare the video files. A custom character was designed for the app using Adobe Illustrator cc 2017 to illustrate the physical activity and movements required in the video files and use a single character in all parts of the application. At first, the custom character was designed, and the 12 exercises movements for the video files were implemented using Adobe After Effect cc 2017 and the character rigging technique on the designed character. In addition, Avidemux 2.7 software was used to edit and resize the video files. The audio files were recorded using recorder and were edited using two software; mp3cutter Pro v3.0.0 and Adobe Audition CC 2017 v10.0 × 64. Eventually, selecting the appropriate font was one of the steps taken to create a good and user-friendly look for the application.

### App coding

The MS self-management application (IDoThis) was developed using the Android Studio environment and Java programming language. The app works on devices running the Android operating system version 4.4 to 12. The application was developed in the Persian language, which was selected according to the geographic area. Of course, developing the app in English is also possible, and the researchers plan to provide the app in English in the future.

### Testing accuracy and performance of the app

The researchers (RF, ZS) tested the developed app performance separately on several runs, and the bugs encountered at each stage were reviewed and corrected. Performance testing was performed for the app. The performance test of an application or system is exercised by emulating actual users [39]. In each test turn, the app was installed and used by two researchers (RF, ZS) and two independent software developers for two to three weeks. The application's performance was evaluated in different sections. The functions of the reporting section of the app were tested and corrected several times to provide accurate, precise, simple, and understandable charts for users. As a user, each member of the evaluation team recorded data in the app, extracted various reports of the recorded data, and tested the accuracy of the reports provided by the app in each test turn.

### Phase 3: App evaluation from perspectives of the end-users

The developed app was evaluated from two aspects: the app's impact on the MS self-management tasks and the app's user-friendliness from the users’ point of view. Twenty participants who were available and willing to cooperate assessed the app. Individuals were eligible to participate in this study if they had been diagnosed as having MS, had an Android smartphone, have the ability and literacy to use applications, and had no cognitive impairment. It should be noted that the participants’ cognitive eligibility was evaluated by the neurologist (SS) in our research team for inclusion in the study. The sampling method for selecting the participants was the snowball sampling method. The app was used for three weeks by the participants. Then their comments were obtained using a researcher-made questionnaire. To facilitate the use of the app, the researchers handed the app guide to each participant in a PDF format and an audio file. After one week from the app installation for each user, the researchers made a telephone call to ask about the use of the app and any need to check and resolve possible issues of the users with the app. Three weeks after the app installation for each participant, a manual or electronic questionnaire was administered to the user based on each participant's convenience. After collecting the users' views, the data were analyzed using the SPSS software (version 24).

The responses to the questions in the questionnaire were based on the Likert five-point scale. The questionnaire validity was assessed by obtaining the opinion of five experts (Health information management field, medical informatics field, and MS fields). Two relative coefficients of content validity ratio (CVR) and content validity index (CVI) were used to check content validity quantitatively.

The content validity ratio (CVR), an item statistic originally suggested by Lawshe (1975), is one of the most widely used methods for quantifying content validity. The panel of experts in the CVR approach is asked to rate each item into one of three categories: “Essential”, “Useful, but not essential”, or “Not necessary”. Items considered “essential” by a critical number of experts are then included in the final form, while items failing to reach this critical level are rejected. CVR is calculated by using this formula: CVR = (Ne − N/2)/(N/2), where Ne is the number of panelists indicating an item as “essential” and N is the total number of panelists [40, 41]. In the present study, all items had a CVR > 0.99 (this value is based on the total number of experts, N = 5, and the numerical values of the Lawshe table) [40]. Therefore, all items were included in the final questionnaire.

CVI is calculated by summing up the agree scores for each item that scored "relevant but needs revision" and "completely relevant" divided by the number of experts. The content validity is confirmed if the CVI score is higher than 0.79, [41]. The CVI was equal to 0.98 in this research.

Cronbach's alpha was calculated separately for two parts of the questionnaire (including the user's perspective on the app's effectiveness in MS self-management and user-friendliness). Cronbach's alpha value was obtained as 0.95 for questions on app effectiveness and 0.64 for user-friendliness questions. Also, alpha was calculated for the sub-sections of the effectiveness section. The alpha values were for the questions in the sub-sections of awareness, behaviour and MS control as 0.86, 0.84 and 0.91, respectively.

The questionnaire consisted of four main sections:1) participant demographic information, 2) App user-friendliness questions, 3) application impact questions, and 4) User comments and Application score (a score between 1 and 5 that each participant considers for the app). The user-friendliness of an application means that the user can easily learn how to work with and use it; overall, if the app is user-friendly, the user has not had to go through a long process to do a simple task [42]. The app's user-friendliness was evaluated with six questions based on a five-point Likert scale ranging from 1 (very difficult or inappropriate) to 5 (very easy or appropriate). App user-friendliness questions were set about how easy it is to install, log in, learn, use, and access the app sections. The impact of app usage was evaluated with 11 questions based on a five-point Likert scale that focused on increasing user awareness in the four dimensions of self-management intended for the app and its impact on changing user behaviour in each of these dimensions. The fourth section of the questionnaire was intended to receive users' feedback on their experience after using the app. In this section, it is possible to express users' opinions about the advantages and disadvantages of the app, prioritize the app modules according to user satisfaction, and users suggestions to improve the app. The qualitative analysis was done for users' comments analysis. The questionnaire is provided in Additional file 2.

## Results

**Fig. 2:**
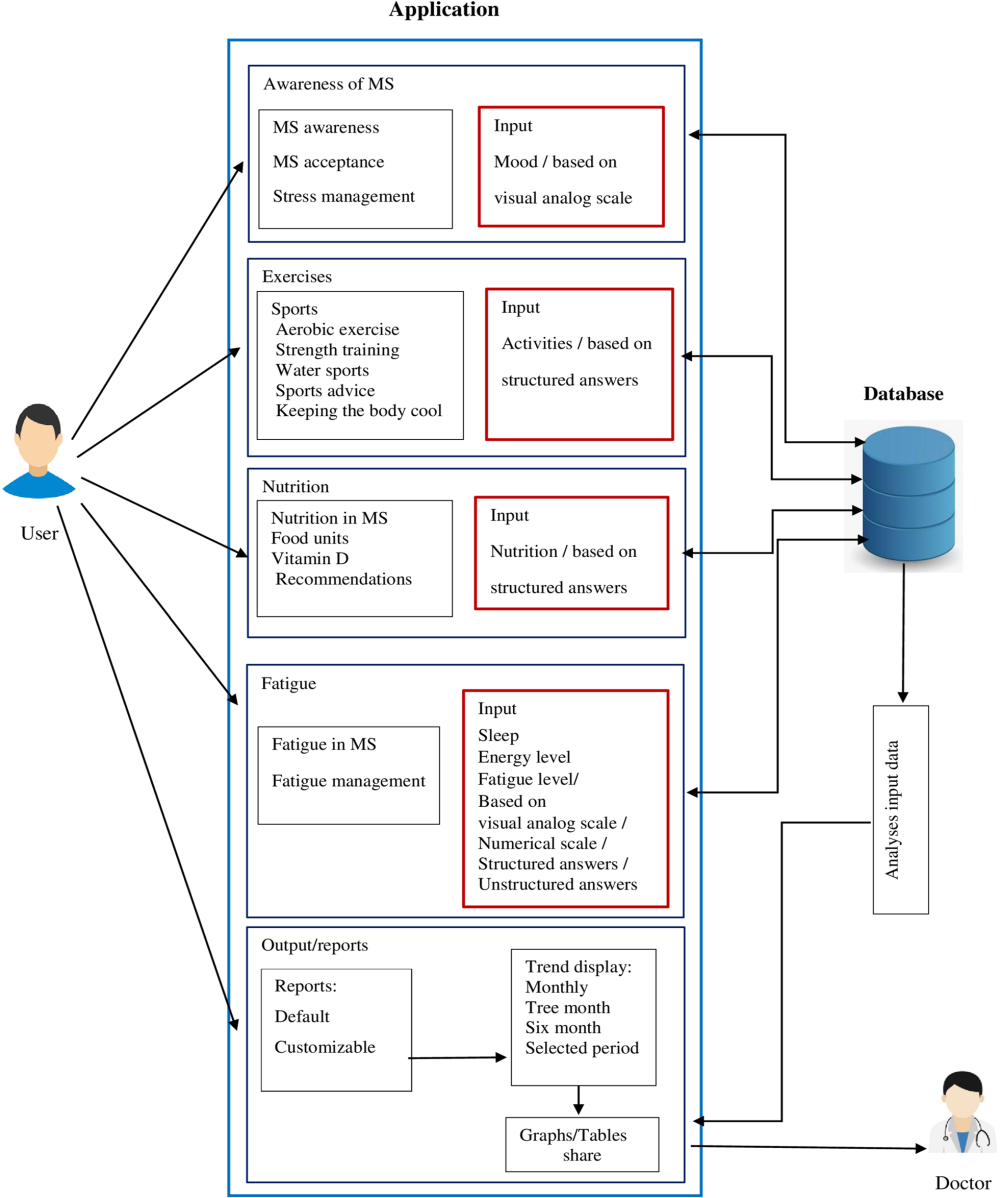
The framework of functionalities of the MS self-management app (IDoThis)

IDoThis app focused on four dimensions among the various dimensions of MS self-management. The dimensions include awareness about MS, healthy living in MS (nutrition and exercise in MS), managing fatigue, and MS monitoring. After preparing the app content, the researchers developed the app's framework of functionalities, as shown in Fig. 2.

### Modules of the app

**Fig. 3:**
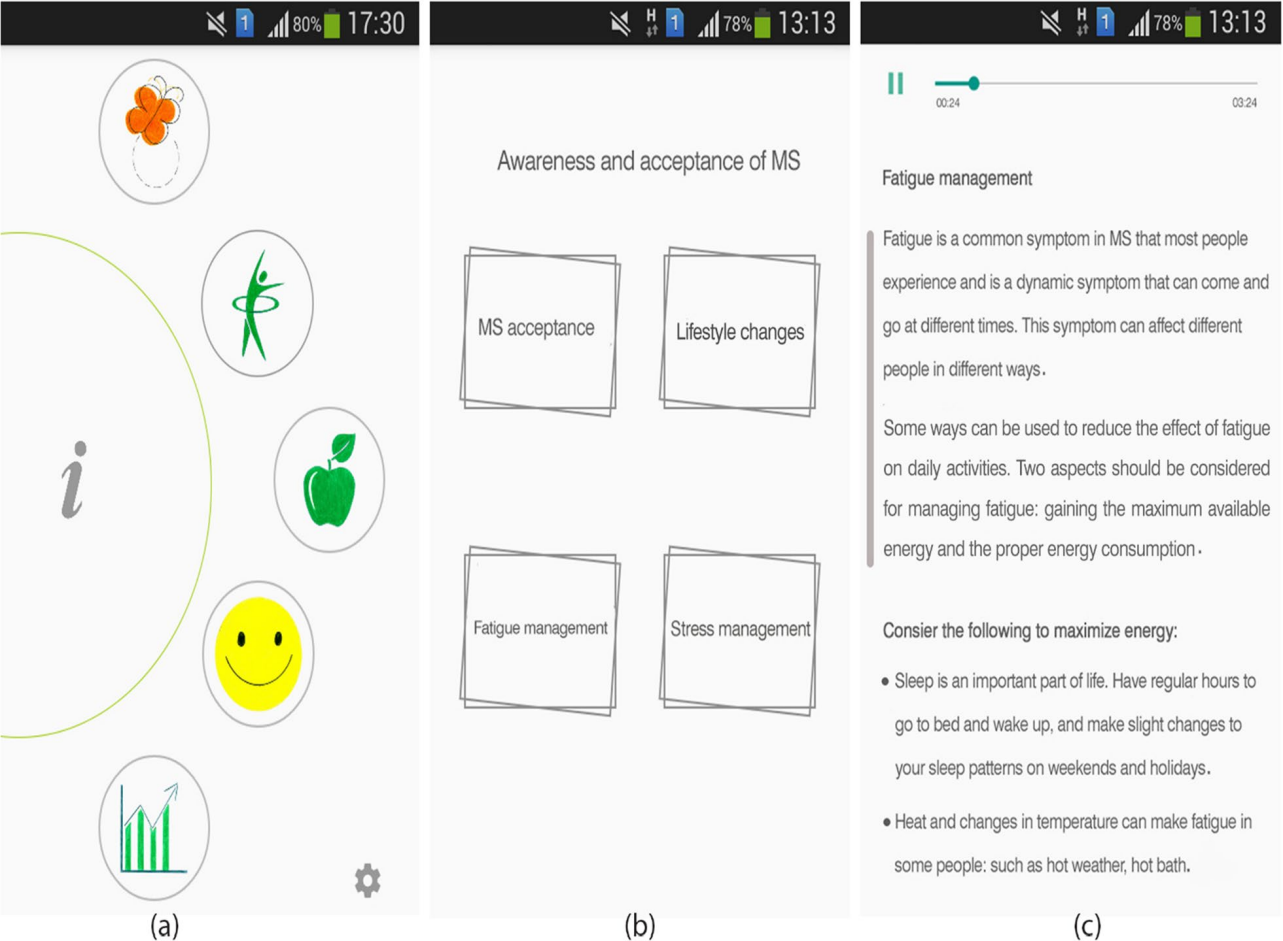
Sample screenshots from IDoThis app. **a** Five modules of the app, **b** Module of MS awareness and acceptance **c** fatigue management subsection

The app was developed around five core modules (Fig. 3):

- three modules for training or informing users (MS awareness, MS nutrition, sports in MS)
- one module for user MS condition monitoring (to enter the user's data for daily monitoring)
- a reporting module (to get the output of the recorded data)

Details of each module and the selected screenshots are presented as follows. The screenshots are shown in the English to understand the present paper better.

1. Awareness and acceptance of MS: To display this section on the main menu, an orange butterfly image has been used as one of the symbols of awareness of MS. The content of this part is presented in four subsections: MS acceptance, lifestyle change, fatigue management, and stress management with text and audio formats (Fig. 3).
2. Exercises in MS: We used an aerobic exercise image to display this section on the main menu. The content is presented in six subdivisions: sports, aerobic exercise, strength training activities, water sports, sports advice, and keeping the body cool through text, audio, and video formats (Fig. 4). This module intends to present helpful exercises for MS patients, the intensity and duration of the exercises, which are recommended in the guidelines and guide, in the form of video files in an attractive way.

**Fig. 4:**
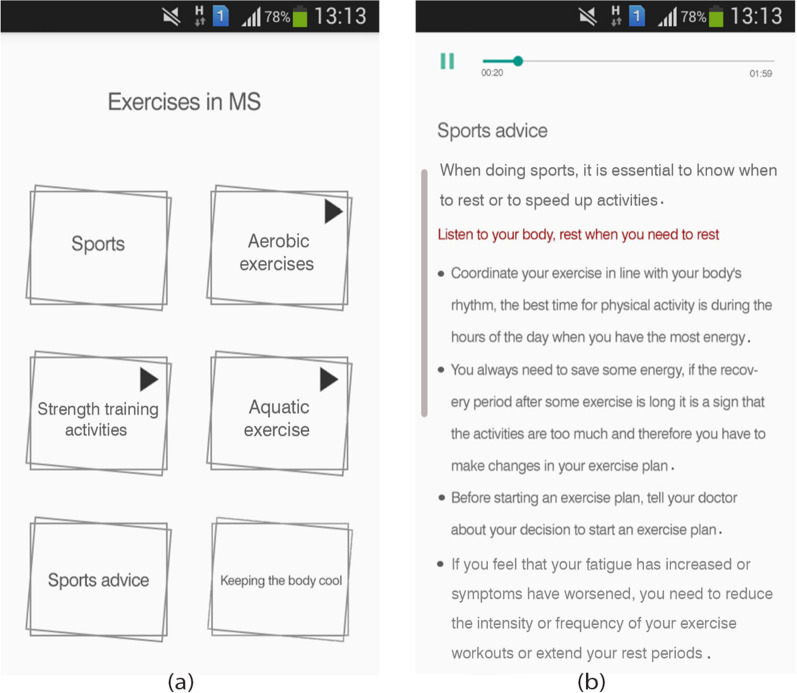
Sample screenshots from IDoThis app (module of exercises in MS). **a** Six subsections of the module, **b** Sports advice subsection
3. Nutrition in MS: We used a green apple image to display this section. The content is presented in four subsections: nutrition in MS, food units, vitamin D, and nutritional recommendations through text, audio, and video formats (Fig. 5). The nutritional recommendations subsection presented in video format, gives practical nutritional recommendations for MS patients based on guidelines.

**Fig. 5:**
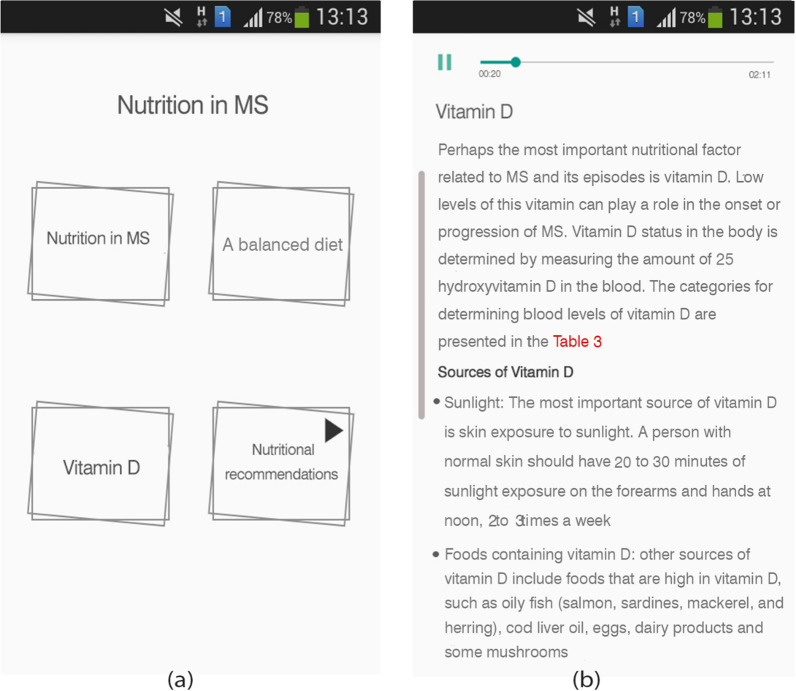
Sample screenshots from IDoThis app (module of Nutrition in MS). **a** Four subsections of the module, **b** Vitamin D subsection
4. Monitoring of MS status: This section aims to monitor the user's MS status in terms of mood status, energy level, sleep, activities & exercises, nutrition, fatigue, and other MS symptoms (Fig. 6). This module allows users to record their mood, energy level, sleep quality and causes of sleep disorders, activities and exercises, compliance with nutritional recommendations, fatigue level (based on a visual analog scale), and other symptoms of MS. In this module, users can also record diaries and search for previously recorded data in terms of date.

**Fig. 6:**
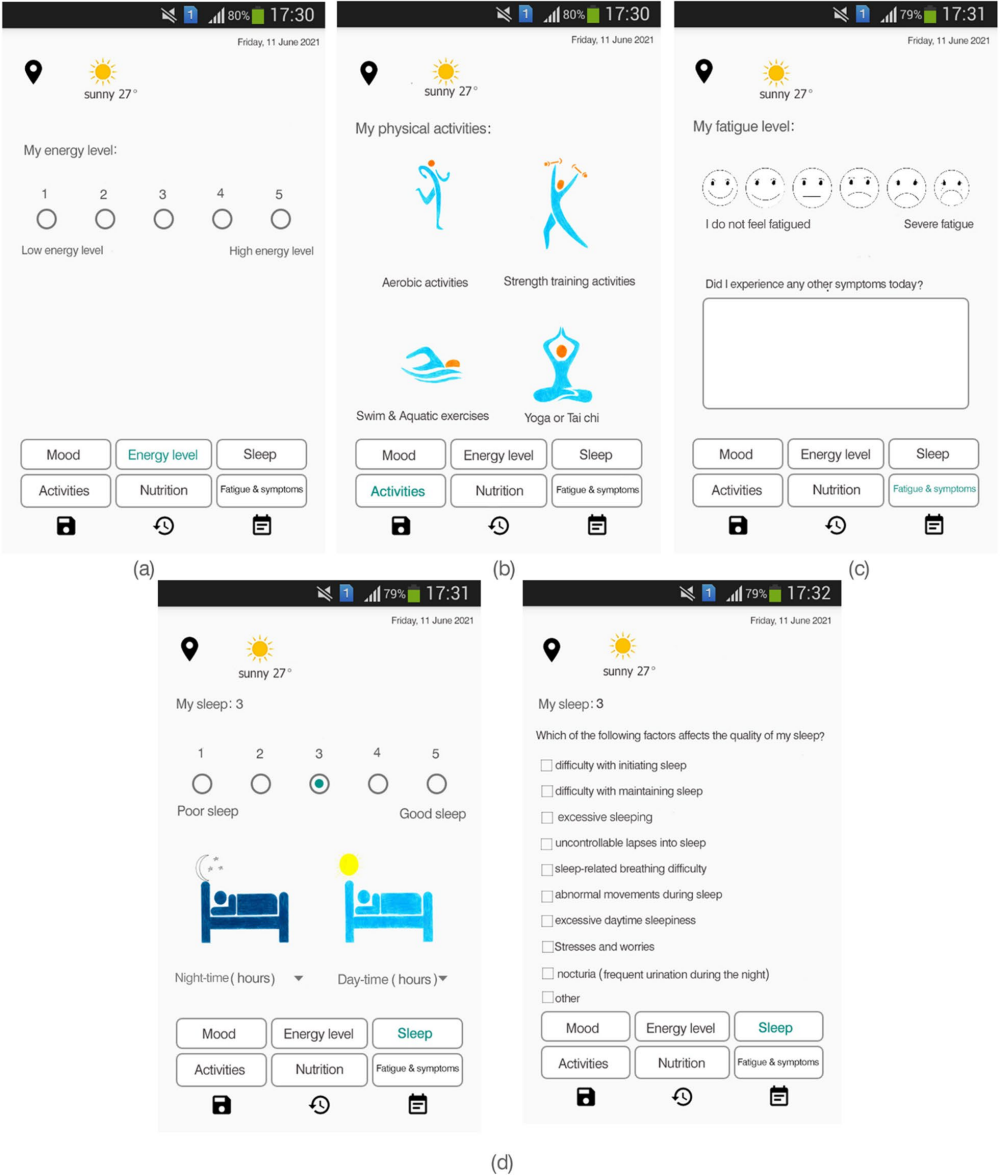
Sample screenshots from IDoThis app (module of the monitoring of MS status). **a** Energy level section, **b** Daily activities section **c** Fatigue section **d** Sleep section (sleep quality and causes of sleep disorders)
  A visual analogue scale was used to determine the level of fatigue in the MS monitoring module (Fig. 6). There are options to record other MS symptoms in text form, given the wide range of MS symptoms each person may experience [43, 44]. The sleep monitoring section makes it possible to score sleep quality, record the hours of sleep at night and during the day, and determine the causes of sleep disorders (Fig. 6).
5. Reporting module: It is possible to report the recorded data by default and in customized way (Fig. 7). In this section, the user can receive understandable and straightforward graphs from the recorded data for each variable. It is possible to extract reports for all variables recorded in the module of MS status monitoring at regular intervals. Users can compile their health data into valuable reports sharable with healthcare providers. One of the features of the reporting module is the possibility of customizing the reports. In addition, it is possible to convert the prepared reports to PDF format and share them.

**Fig. 7:**
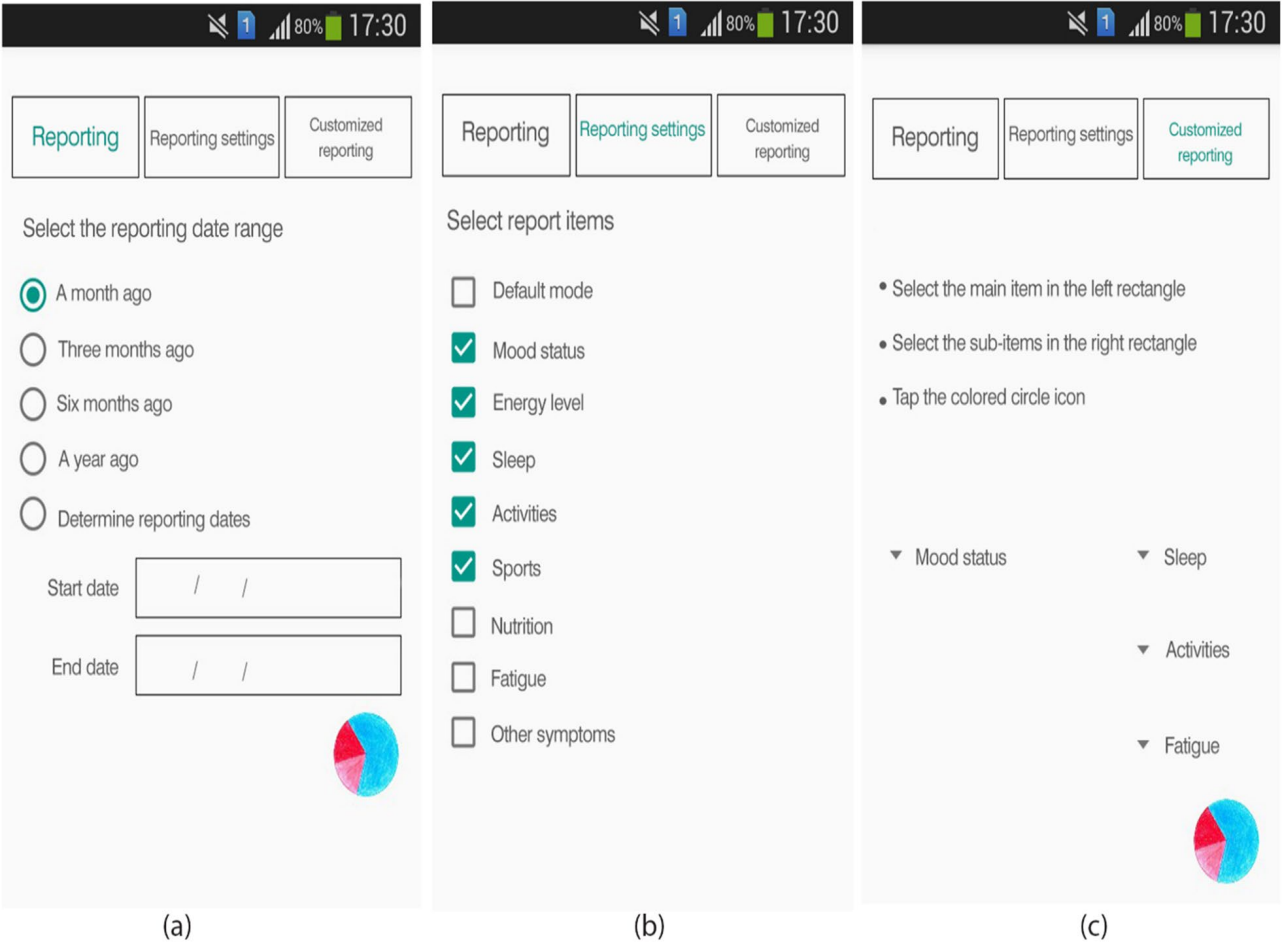
Sample screenshots from IDoThis app (module of reporting). **a** Reporting section, **b** Reporting setting section **c** customized reports section

**Fig. 8:**
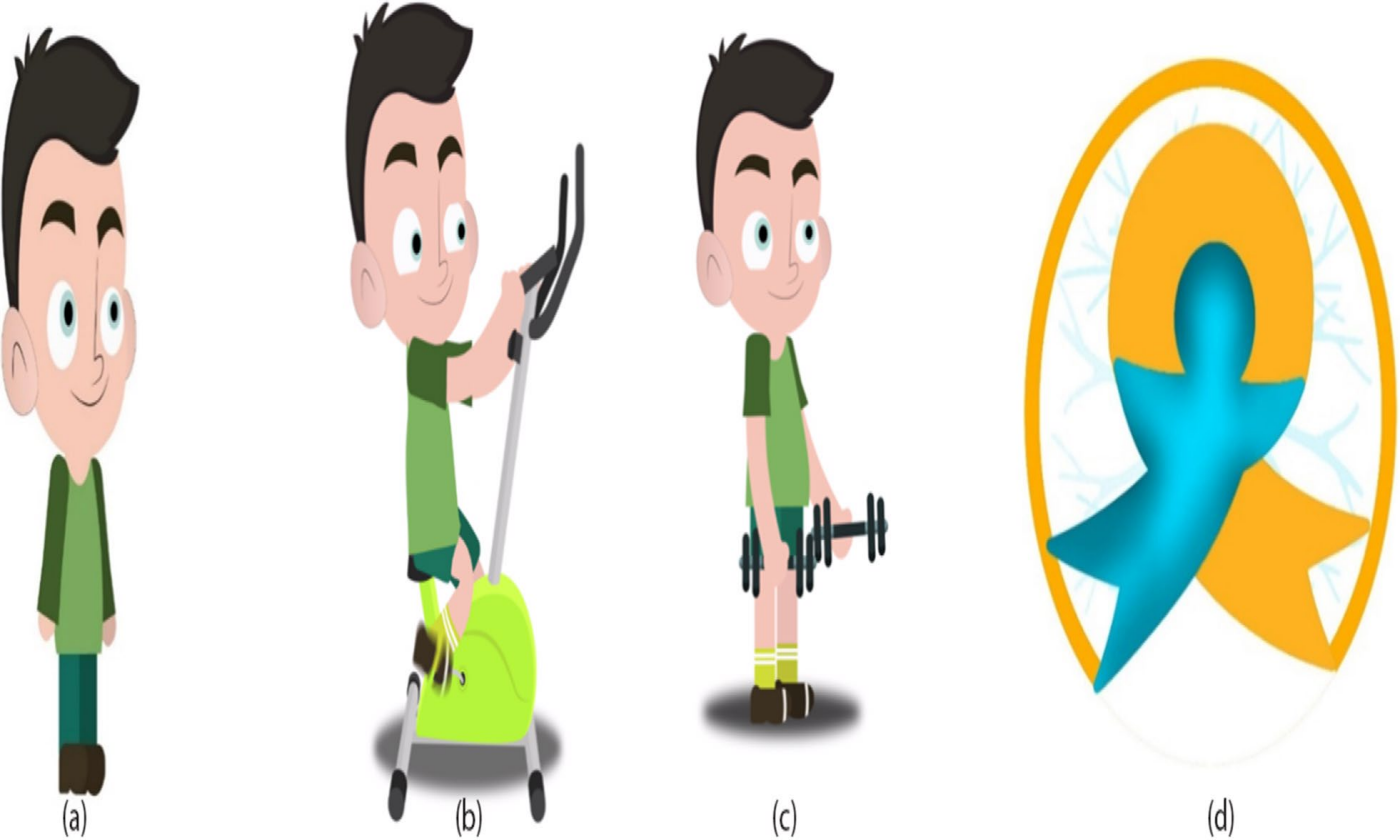
Sample images from the IDoThis app elements. **a** The custom character, **b**, **c** Two examples of exercises movements in video files, **d** The final logo of the app

Texts, images, audio files, and video files have been used to provide content in the app. According to the previous explanations, a unique character was designed for the app. The desired movements were prepared using this character for the video files in the exercise and nutrition sections. The image of the unique character and the final app logo can be seen below (Fig. 8).

### App evaluation from perspectives of the end-users

40% of the participants were educated to a bachelor's degree and 25% to a master's degree. 45% were housekeepers, and 35% had full-time job. 60% of participants were lived < 6 years with MS, and 35% had lived 6–10 years with MS.

According to the findings, 85% of the users stated that they would recommend this application to others. 40% of the participants assigned the app a score of 4, and 25% assigned a score of 5 from the range of 1 to 5.

75% of participants mentioned that using this app improved their MS self-management status at intermediate and higher levels, but 25% of the participants mentioned that the app had no or very low effect on improving their MS self-management. The users’ perceptions about the app's effectiveness for each item of the MS self-management tasks are listed in Table 1.

**Table 1:** The users’ perspectives on the app's short-term impact and the user-friendliness of the app

Number	Effectiveness items	Not at all (%)	Slightly (%)	Moderately (%)	Very (%)	Extremely (%)
1	Using the app improved my awareness of MS self-management ways.	5	0	45	40	10
2	Using the app improved my awareness of MS proper nutrition.	10	5	35	35	15
3	Using the app improved my awareness about useful exercise in MS.	10	5	35	20	30
4	Using the app helped me to follow nutrition recommendations.	15	20	20	40	5
5	Using the app helped me to do useful exercises.	10	10	50	30	0
6	Using the app improved my motivation to monitor my MS daily.	10	0	65	20	5
7	Using the app helped me to monitor my sleep.	25	15	40	20	0
8	Using the app helped me to monitor my energy level.	10	20	40	30	0
9	Using the app helped me to monitor my fatigue level.	25	10	30	25	10
10	Using the app helped me to remember my MS symptoms and share them with the physician.	25	5	25	35	10
11	Using the app improved my MS management.	10	15	50	25	0

Number	User-friendliness items	Not at all (%)	Slightly (%)	Moderately (%)	Very (%)	Extremely (%)
1	Using the app was easy.	0	0	25	50	25
2	Installing the app was easy.	0	0	10	55	35
3	Entering the app was easy.	0	0	15	60	25
4	The format of content presentation in the app was appropriate.	0	5	25	50	20
5	Learning to use the app was easy.	0	0	35	40	25
6	Accessing to different sections of the app was easy.	0	0	5	50	45

The majority of the users rated the user-friendliness of the app as high. The percentage of the user-friendliness of the app for each item is listed in Table 1.

## Discussion

MS self-management is a complex, personalized, and ongoing process. MS self-management includes both elements of the general MS self-management framework and involves paying particular attention to the specific needs of each individual with the unique experience of living with MS in each person [7, 8]. The present study considered these two elements for developing the app. To address the general dimensions of self-management, the researchers provided the required information for managing MS in app's three modules (MS awareness, MS nutrition, and exercises in MS). Considering the nature of MS and the broad spectrum of its related problems in each individual, the daily status monitoring module, and the reporting module were provided in the app.

The MS monitoring module focuses on mood, energy, sleep, activity, nutrition, fatigue, and MS symptoms to identify the user's MS status. Most of these elements are available in some MS self-management apps, such as the MS assistant app and MS self app [33, 45]. However, some other apps for MS self-management focused on describing the patient's condition (symptoms and attacks), managing medications, calculating scales of depression and anxiety, calculating the severity of fatigue, and the relationship between physician and patient [46].

The IDoThis app on fatigue dimension provided the general explanations of fatigue and standard fatigue management approaches such as saving energy, prioritizing and simplifying tasks, identifying the level of fatigue, and identifying various factors that affect fatigue. However, there are dedicated apps for MS fatigue management with a deeper look at fatigue management and its control strategies, such as the MS Energize app. In this app, fatigue management approaches and information have been presented in a more interactive environment. As such, it attracts users and results in positive feedback [47]. Alternatively, another MS Fatigue app focuses more on assessing fatigue, fatigue-related mood symptoms (e.g. depression and anxiety), and pain [48].

A significant percentage of participants (70%) reflected their satisfaction with sections "Exercises in MS" and "Daily monitoring" in the app. Physical activity and exercise are important factors for improving and managing the physical demands of MS patients. A systematic study in 2018 stressed the importance of having exercise programs and highlighted it among unmet needs in existing MS apps [23]. Moreover, lack of enjoyment in physical activities is considered a barrier that decreases the motivation for exercising in persons with MS [23, 49]. Therefore, providing information on physical activities and beneficial sports in MS, exercise recommendations, level and intensity of these activities based on the guidelines can explain users' satisfaction with the "exercise in MS" module in the app. Considering video, audio, and text formats for presenting information in this module might contribute to this section's user motivation, fun, and satisfaction, since the inclusion of multimedia content is highly recommended in mobile apps [50].

Daily monitoring of the MS status can help identify the unique aspects of MS in each person and encourage the user to pay special attention to MS [51]. According to the participants' statements, the MS status monitoring module was the second most crucial part of the app from the participants' perspective. This module encompasses components such as" monitoring mood," "monitoring energy levels and fatigue," "monitoring sleep patterns and causes of sleep disorders," "monitoring daily activities," " monitoring exercises," and " monitoring nutritional status." Users' satisfaction with the MS status daily monitoring module can be due to the possibility of recording data, reporting the recorded data, customizing report items, and as a result, recognizing more about MS in each person (considering the wide variety of MS symptoms between among patients).

Energy conservation and fatigue management are two fundamental self-management needs of people with MS and essential features in developing health solutions for MS patients. Appropriate information and tools can help users manage their daily activities toward meeting these needs [49, 52–54]. Information on energy management, fatigue management, and strategies for optimal energy consumption has been presented in the patient education module in our app. In addition, the possibility of recording sleep quality, exercises, daily activities, and fatigue levels for setting and managing activities has been provided in the IDoThis app. According to the evaluation results of the users' comments, the app failed to satisfy users in terms of helping them control sleep, optimize energy consumption, and manage fatigue. However, it appears to moderately motivate users to monitor sleep status, energy level, and fatigue level. Users' dissatisfaction in these dimensions can be due to needing to meet users' expectations with these sections. According to users’ comments in the fourth part of the questionnaire, the lack of serious attention to fatigue management solutions, ways to improve sleep status, and optimal energy consumption strategies in the app, have caused them to be dissatisfied with these sections. None of the participants mentioned any usability issues.

Another item in the daily monitoring module is "monitoring sleep patterns and causes of sleep disorders." The prevalence of sleep disorders is significantly higher in people with MS than in the general population, and more than half of people with MS have significant sleep disturbance [55–57]. Sleep disturbance is a general term for a wide range of sleep-related symptoms in people with MS that may include: difficulty with initiating or maintaining sleep (insomnia), too much sleep (hypersomnia), difficulty controlling sleep or sleep attack (narcolepsy), sleep-related breathing difficulty, abnormal movements during sleep such as restless legs syndrome, abnormal behaviors during sleep, and excessive daytime sleepiness [55–60]. Helping to identify daily sleep status and user's sleep difficulty in the IDoThis app attracted users' attention of users in this project. However, the users were dissatisfied due to the lack of adequate solutions to manage sleep status in the app. Due to the wide range of sleep disturbances in MS patients and different causes (such as secondary effects of common MS symptoms, side effects of MS medications, reduced physical activity, effects of stress and anxiety, effects of depression, changes in sleep hygiene behaviors, and other factors) that can be lead to these disturbances. Therefore, to improve and manage patients' sleep disorders, pharmacological and non-pharmacological interventions are performed by considering the type of sleep disorder and its causes [57, 61]. As a result, providing solutions and strategies to improve sleep status in users requires an in-depth review of guidelines. Considering the four dimensions of the app and the volume of app content, the researchers decided to focus only on identifying the amount of sleep and sleep disorders of users in the monitoring module. Although the failure to meet users' expectations in terms of the sleep section was considered one of the weaknesses of the current app, the suggestion to develop dedicated apps to identify and manage sleep disorders in MS patients can be a more practical solution. In addition, dedicated apps for fatigue management should be developed, as fatigue in MS patients can be directly due to mechanisms of MS disease or the consequence of various secondary causes such as mood, depression, stress, sleep disorders, pain, muscle weakness, temperature, or side effects of medications. Fatigue in people with MS is considered highly individual [62, 63].

This study had several limitations. Twenty participants who were available and willing to cooperate with the researchers assessed the app. Although according to some studies, this size of participants in usability tests can detect more than 80% of the problems [64, 65], the number of participants in our initial evaluation may not been enough due to the wide range of changes in the physical abilities of people with MS [66]. In future research, we should consider a large size of sample for a more extended period of follow-up. The missing of information on the MS disease courses and EDSS (Expanded Disability Status Scale) of participants was another limitation of this study.

In some dimensions, the app only measures and identifies that aspect and does not deal with the management and improvement strategies, that sleep and fatigue are two of these aspects. The various causes of sleep disorders in MS patients, the need for an in-depth survey of different sleep improvement strategies, different levels of fatigue, and various factors affecting fatigue in each patient, the need for an in-depth survey of varying fatigue management strategies in MS patients were the main reasons for the researchers' decision not to enter the profound aspects of sleep disorders and fatigue management in this app. Thus only monitoring and identifying the sleep disorders factors were included in the app. Moreover, in the fatigue dimension, the app focused solely on increasing users' awareness of general fatigue management strategies, monitoring the fatigue level, and recognizing the affecting factors of fatigue.

Another limitation of the current study was that the questionnaire used to evaluate the application did not include questions about the layout of the application. Web Content Accessibility Guidelines (WCAG) were not considered during development. The developed app is the initial version, and we will consider WCAG as one of the requirements in the following updates and future versions of this app. In addition, the app is currently only developed in Persian language and for the Android operating system, which iPhone users cannot use. Providing English language and the possibility of using the app for iPhone users will also be considered in the developing the next versions of the app.

## Conclusion

The present app is a self-management app for MS patients developed in Persian. The findings showed that the app could satisfy users, help them become aware of some self-management aspects of MS and monitor their daily status. The results also indicate that the users’ satisfaction of MS self-management apps is directly related to the extent to which the needs of people with MS are met and the possibility of customizing applications. The complex and unique nature of MS and the emergence of different range of symptoms in each person have made it impossible to develop a comprehensive application to cover all aspects of MS self-management. Therefore, the development of specific apps for a few dimensions of MS self-management or for management of several symptoms from the wide range of MS symptoms or developing apps considering the different range of ability levels in people with MS can be more practical and constructive strategy for encouraging individuals with MS to use MS self-management apps. Given the evolution process of apps, the developed app could be a starting point for developing better apps for MS self-management in future studies.

In the next stages of application development, the researchers plan to update the content of the current application's content to meet the users’ unmet expectations. The researchers plan to conduct more detailed research on the three dimensions of fatigue, stress and sleep in MS toward developing a dedicated app for each of the above dimensions.

## Supplementary Information

**Additional file 1**. The list of all selected guidelines and guides for the app content preparation.

**Additional file 2**. The app evaluation questionnaire from users' perspectives.

## Data Availability

The data that support the findings of this study are available from the corresponding author on reasonable request.
