# Supplementary material for: A mobile app (IDoThis) for multiple sclerosis self-management: development and initial evaluation
Source: BMC Med Inform Decis Mak. 2022 Dec 13;22:328. doi: 10.1186/s12911-022-02078-z (PMC9745928; doi:10.1186/s12911-022-02078-z)
Supplement: Supplementary file 1 — Additional file 1. The list of all selected guidelines and guides for the app content preparation. [file 12911_2022_2078_MOESM1_ESM.pdf]

The list of all the selected guidelines and guides for app Content preparation

**guidelines and guides**	**Publisher**
MS in focus - Is it MS?	Multiple sclerosis international federation
MS in focus – Information, Technology and MS	Multiple sclerosis international federation
Living Well With MS	Multiple Sclerosis Trust
MS and Life Choices	Multiple Sclerosis Trust
MS and your feelings	Multiple Sclerosis Trust
MS in focus – Healthy Living	Multiple sclerosis international federation
Wellness Discussion Guide for people with MS and Their HealthCare Providers	National Multiple Sclerosis Society
Wellness for people with MS: What do we know about Diet, Exercise and Mood and what do we still need to learn?	National Multiple Sclerosis Society
Living with fatigue- fatigue management for people with MS	Multiple Sclerosis Trust
MS in focus – Fatigue and MS	Multiple sclerosis international federation
Taming Stress in Multiple sclerosis	Multiple Sclerosis Society of Canada
SLEEP DISRTIBUTION AND MULTIPLE SCLEROSIS	National Multiple Sclerosis Society

**guidelines and guides**	**publisher**
MS and me: a self-management guide to living with MS	Multiple Sclerosis Trust
Diet	Multiple Sclerosis Trust
Vitamin D	Multiple Sclerosis Trust
Healthy Eating: A guide for people with MS	Multiple Sclerosis Society of Canada
DIET AND MULTIPLE SCLEROSIS	National Multiple Sclerosis Society
National Guideline in Multiple Sclerosis	The MS Society of Western Australia
AQUATIC EXERCISE & MULTIPE SCLEROSIS: A Guide for patients	Multiple Sclerosis Association of America
Exercise as Part of Everyday Life	National Multiple Sclerosis Society
Physical Activity - GUIDE FOR PEOPLE WITH MS	Multiple Sclerosis Society of Canada
Canadian Physical Activity Guidelines: for adult with multiple sclerosis	Multiple Sclerosis Society of Canada
Multiple Sclerosis and exercise	Exercise in Medicine Australia
Aquatic Exercise and Multiple Sclerosis	MS Australia
Fitness Exercise and Multiple Sclerosis	MS Australia
Multiple Sclerosis and exercise	MS Australia
