# Supplementary material for: A mobile app (IDoThis) for multiple sclerosis self-management: development and initial evaluation
Source: BMC Med Inform Decis Mak. 2022 Dec 13;22:328. doi: 10.1186/s12911-022-02078-z (PMC9745928; doi:10.1186/s12911-022-02078-z)
Supplement: Supplementary file 2 — Additional file 2. The app evaluation questionnaire from users' perspectives. [file 12911_2022_2078_MOESM2_ESM.pdf]

The App evaluation questionnaire from users' perspectives

The "IDO THIS" app is developed to help MS self-manage. The present questionnaire has been prepared to evaluate the user-friendliness and short-term impact of this app. This questionnaire will be provided to you after three weeks of installing the app on your mobile phone and using it, that us to receive your comments and evaluation of the app.

Section A: Demographic information

1.Gender: ☐Female ☐Male

2.Age:

3.Education level:

☐Literacy ☐Elementary ☐Diploma

☐Associate degree ☐Bachelor's degree ☐Master's degree and higher

4.Working status:

☐Unemployed ☐Student ☐housewife ☐Retired ☐Employed

5.How many years have you had MS? ………………year/years

Section B: User-friendliness of the app

1. How do you evaluate the ease of using the app?

Very difficult				Very easy
1	2	3	4	5

2. How do you evaluate the ease of installing the app?

Very difficult				Very easy
1	2	3	4	5

3. How do you evaluate the ease of entering the app?

Very difficult				Very easy
1	2	3	4	5

4. How do you evaluate the presentation of information in the app?

1 Not at all appropriate

2 Not very appropriate

3 Somewhat appropriate

4 Appropriate

5 Very appropriate

5. How do you evaluate the ease of learning to use the app?

Very difficult				Very easy
1	2	3	4	5

6. How do you evaluate the ease of access to different parts of the app?

Very difficult				Very easy
1	2	3	4	5

Section C: Impact of the app

1. What effect did using the app have on your knowledge of MS self-management methods?

1 Did not increase my awareness at all

2 Increased my awareness a little

3 Increased my awareness moderately

4 Increased my awareness good

5 Increased my awareness a lot

2. What effect did the app have on your awareness of proper nutrition in MS?

1 Did not increase my awareness at all

2 Increased my awareness a little

3 Increased my awareness moderately

4 Increased my awareness good

5 Increased my awareness a lot

3. What effect did the app have on your awareness of the beneficial exercises in MS?

1 Did not increase my awareness at all

2 Increased my awareness a little

3 Increased my awareness moderately

4 Increased my awareness good

5 Increased my awareness a lot

4. What effect did the app have on the observance of nutritional recommendations (compared to before using the app)?

1 Had no effect on adherence to recommendations

2 Had little effect on adherence to recommendations

3 Helped me to follow some recommendations

4 Helped me follow rather much recommendations

5 Helped me follow all of the recommendations

5. What was the effect of using the app in doing sports activities by you?

1 Did no effect on doing sports activities

2 Had little effect on doing sports activities

3 It caused that sometimes, I do some sports activities

4 It caused most of the time, I do some sports activities

5 It caused regularly, I do some sports activities

6. How did the app affect your motivation to monitor your MS daily?

1 Did no effect on my motivation

2 Had little effect on my motivation

3 It increased my motivation moderately

4 It increased my motivation rather much

5 It increased my motivation highly

7. What effect did the app affect your sleep status control?

1 It did no effect

2 It had little effect

3 It had a moderate effect

4 It had a rather much effect

5 It had very much effect

8. What effect did the app have on your energy level control?

1 It did no effect

2 It had little effect

3 It had a moderate effect

4 It had a rather much effect

5 It had very much effect

9. What effect did the app have on your fatigue level control?

1 It did no effect

2 It had little effect

3 It had a moderate effect

4 It had a rather much effect

5 It had very much effect

10. What effect did the app in reminding your MS symptoms and sharing them with your physician?

1 It did no effect to remember of my MS symptoms

2 It had little effect to remember of my MS symptoms

3 It effected to remember of some my MS symptoms

4 It effected to remember most of my MS symptoms

5 It effected to remember all of my MS symptoms, exactly

11. Eventually, what effect did the app have on your MS self-management?

1 It did not affect improving my MS self-management

2 It had little effect improving my MS self-management

3 It had somewhat effect improved my MS self-management

4 It had a rather much effect improved my MS self-management

5 It had very much effect improved my MS self-management

Section D: user comments & application score

1. Would you recommend this app to people who might benefit from it?

☐Yes

☐No

2. What is your overall score for the app (from one to five)?

☐1 ☐ 2 ☐3 ☐ 4 ☐ 5

3. If you want to consider the different parts of the app as its strengths and weaknesses, prioritize these parts from strong to weak, respectively.

A. Aware of MS

B. Exercise in MS

C. Nutrition in MS

D. Daily status monitoring

E. Reports

strong	
↓	
weak	

4. What do you think are the shortcomings of the app that should be addressed in future updates?

…………………………………………………………………………………………………….

…………………………………………………………………………………………………….

…………………………………………………………………………………………………….

5. Suggestions and Comments:

……………………………………………………………………………………………………

……………………………………………………………………………………………………

…………………………………………………………………………………………………….

Thanks
